# Role of TGF-****β**** Pathway Polymorphisms in Sporadic Thoracic Aortic Aneurysm: rs900 TGF-****β****2 Is a Marker of Differential Gender Susceptibility

**DOI:** 10.1155/2014/165758

**Published:** 2014-02-24

**Authors:** Letizia Scola, Federica M. Di Maggio, Loredana Vaccarino, Manuela Bova, Giusy I. Forte, Calogera Pisano, Giuseppina Candore, Giuseppina Colonna-Romano, Domenico Lio, Giovanni Ruvolo, Carmela R. Balistreri

**Affiliations:** ^1^Department of Pathobiology and Medical and Forensic Biotechnologies, University of Palermo, Corso Tukory 211, 90134 Palermo, Italy; ^2^Unit of Cardiac Surgery, Department of Surgery and Oncology, University of Palermo, Palermo, Italy

## Abstract

Thoracic aortic aneurysm (TAA) is a progressive disorder involving gradual dilation of ascending and/or descending thoracic aorta with dissection or rupture as complications. It occurs as sporadic or defined syndromes/familial forms.Genetic, molecular and cellular mechanims of sporadic TAA forms are poorly characterized and known. Thus, our interest has been focused on investigating the role of genetic variants of transforming growth factor-**β** (TGF-**β**) pathways in TAA risk. On the other hand, no data on the role of genetic variants of TGF-**β** pathway in sporadic TAA exist until now. In addition, other cytokines, including IL-10, orchestrate TAA pathophysiology. Their balance determines the ultimate fate of the aortic wall as healing atherosclerosis or aneurysm formation. Thus, in this paper it was analyzed the role of ten polymorphisms of genes encoding TGF-**β** isoforms and receptors, and IL-10 in sporadic TAA. Our study included cases affected by sporadic TAA and two control groups. The most relevant finding obtained allows us to propose that rs900 TGF-**β**2 SNP is associated with sporadic TAA in women. This might open new perspectives for the analysis of sporadic TAA susceptibility factors and prevention.

## 1. Introduction

Thoracic aortic aneurysm (TAA) is a pathological widening of aorta resulting from degeneration of the extracellular matrix and loss of smooth muscle cells in the tunica media. TAA has different etiological causes, including monogenic syndromes (such as Marfan and Loeys-Dietz syndromes), bicuspid aortic valve (BAV) disease, and idiopathic causes [1, 2].

The pathogenesis of TAA in monogenic syndromes has been extensively studied [3]. The attested evidence, indeed, suggests the deregulation of transforming growth factor-*β* (TGF-*β*) signaling characterized by its enhanced function and damaged TGF-*β* receptors as their common and typical feature [4, 5].

The TGF-*β* family is constituted by TGF-*β*1, TGF-*β*2, and TGF-*β*3 members, which are pleiotropic secreted cytokines having a broad spectrum of biologic functions. Among these, the TGF-*β*1 has numerous cellular functions, including cell growth, cell proliferation, cell differentiation, and apoptosis. In humans, TGF-*β* gene product's effects can stimulate or inhibite cell growth depending cellular and tissue targets. TGF-*β*1 can modulate cell differentiation and proliferation in auto- or paracrine manner [6]. In vascular smooth muscle cells, TGF-*β* can upregulate fibronectin and connective tissue growth factor expression via activation of small mothers against decapentaplegic (Smad) proteins [7]. As a consequence, it can promote the deposition of components of extracellular matrix (ECM) [8]. Furthermore, its action depends on the interaction with specific receptors, such as TGF-*β* receptor (TGF-*β*R) I and TGF-*β*RII, glycoproteins of 55 kDa and 70 kDa, respectively, with core polypeptides of 500–570 amino acids [9].

TGF-*β* actually is considered as a crucial player in vascular remodelling, able to alter both structure and ECM composition. In Marfan syndrome, the fibrillin-1 gene mutations seem to influence the bioavailability of active TGF-*β*. In addition, mutations in the TGF-*β* receptors also impair the signalling cascade in other Marfan syndrome related disorders, including Loeys-Dietz syndrome, familial TAAs, and aortic dissection. Furthermore, mutations in Notch gene homolog 1 (NOTCH1) and Notch1 pathway, mainly identified in TAA patients with BAV, seem to influence TGF-*β* crosstalk [10].

In contrast, molecular and genetics mechanisms of the nonfamilial TAA forms, representing the major number of cases of TAAs, remain largely unknown [11]. Different roles of TGF-*β* pathways in tissue remodelling mechanisms have been reported in both sporadic thoracic and abdominal aneurysms [8]. In particular, both loss and gain of functional TGF-*β* signalling have been described as predisposing factors for both sporadic TAA development and dissection. The paradoxical effect of TGF-*β* leading to enhanced connective matrix degradation through metalloproteinase activation has been principally observed in the nonsyndromic cases of familial TAAs and dissection [4]. In addition, TGFBR1 and TGFBR2 losses induced by functional mutations have been associated with both familial syndromic and nonsyndromic TAAs [12–14]. This altered condition of TGF-*β* signalling has been demonstrated to induce unusually the activation of TGF-*β* mediated connective matrix degradation [4].

Furthermore, vascular remodelling, characterising both sporadic thoracic and abdominal aneurysm, seems prevalently to be the result not only of TGF-*β* pathways, but also of upregulation of multiple cytokines, including interleukin-10 (IL-10), an anti-inflammatory cytokine able to modulate activity of TGF-*β* pathways. A large variety of immune and tissue aorta cells evocate the typical aorta abnormalities of thoracic and abdominal sporadic aneurysms. The balance of cellular type and resultant cytokine milieu determines the ultimate fate of the aortic wall healing, atherosclerosis, or aneurysm formation. In the complex scenario, another crucial factor is the genetics propensity [15, 16]. Polymorphisms of IL-10 gene have been associated with abdominal aneurysms, while no data exist in literature about their role in sporadic thoracic aneurysms [17–20].

Based on these observations, in this paper we sought to analyse the role of some common single nucleotide polymorphisms (SNPs) of genes encoding TGF-*β* isoforms and receptors, and IL-10 and receptor in sporadic TAA. On the other hand, no literature data on the role of genetic variants of TGF-*β* and IL-10 pathways in sporadic TAA exist until now.

## 2. Materials and Methods

### 2.1. Patient and Control Populations

Our study included 144 individuals (107 men (74.3%) and 37 (25.7%) women; mean age: 63 ± 10.7) from Western Sicily enrolled precisely from January 2004 to July 2008 at time of their admission to Cardiac Surgery Unit of Palermo University Hospital. They were affected by sporadic TAA, diagnosed through ECHO, CT, and MRI imaging technologies and with localization essentially in ascending aorta (precisely in aortic sinus and tubular portion and sometimes only in tubular portion) and in aortic bulb, or both (Table 1). Familial and syndromic forms (i.e., Marfan and Ehlers-Danlos syndromes) and autoimmune connective tissue disorders were excluded through histopathological criteria and phenotypic analyses.

Medical histories pertinent to aortic diseases were obtained from patient's medical records. Thus, demographic and clinical features, comorbidity conditions, and pharmacological treatments were collected (Table 1).

To perform genotype analyses two different control populations were also enrolled. The first included 90 unrelated patients of the same cardiac unit without TAA (56 (62%) men and 34 (38%) women; mean age: 61.08 ± 5.83 years). The second control group was represented by 168 healthy control (112 (66.7%) men and 56 (33.3%) women mean age: 45.2 ± 7.44 years). Their demographic and clinical features ECHO imaging exclusion of aorta wall abnormalities, comorbidity conditions, and pharmacological treatments were collected (see Table 1).

Patients and controls belonged to the same ethnic group, since their parents and grandparents were born in Sicily. Healthy control age was significantly lower respect to that of the two groups of patients and hypertension characterised the 79% of all patients, opportunely treated with medications like ACE inhibitors and beta-blocker, and so forth during the follow-up and after surgical procedures (Table 1).

Our study received approval from local ethic committee and all participants gave their informed consent. Data were encoded to ensure privacy protection of patients and controls. All laboratory procedures were performed without knowledge about nature of material.

### 2.2. Molecular Typing

As reported in Table 2, we selected ten functional and common SNPs of IL-10 and TGF-*β* pathways located in the promoter region, codifying and noncodifying sequences and 3′UTR region. Information about these SNPs was acquired from dbSNP NCBI, the ENSEMBL database (http://www.ensembl.org/index.html), and the UCSC Genome Browser website (http://genome.ucsc.edu/). The allelic and genotypic frequencies of TGF-*β* and IL-10 SNP pathways were detected through the assays on demand developed by KBioscience Ltd. (Middlesex, UK) and based on a homogeneous Fluorescence Resonance Energy Transfer (FRET) detection and allele specific PCR (Kaspar). Briefly, two specific oligonucleotides were designed for each allele of the SNPs studied. Each one of these oligos was tailed with an 18 bp sequence distinct from each other. Taq polymerase, dNTPs, an internal standard dye (rhodamine X, Rox), and reverse primers were included. In addition, the KBioscience modified versions of Taq polymerase are unable to extend primers characterised to be mismatched at their 3′ terminal base. This property was used to discriminate the two alleles. The reaction was monitored by the fluorescence signals released by two other FRET reporter oligos included in the reaction mixes. The endpoint fluorescence emission was detected on an ABI-Prism 7300 Real-Time PCR Analyzer (Applied Biosystem, USA). The genotypes were determined using the 7300 system SDS software, versus 1.3 (Applied Biosystems) sample by sample, on the basis of the detection of a unique (homozygous samples) or double (heterozygous samples) fluorescence signals.

### 2.3. Statistical Analysis

Allele and genotype frequencies were evaluated by gene count. Data were tested for goodness of fit between observed and expected genotype frequencies according to Hardy-Weinberg equilibrium, by *χ* ^2^ tests. Significant differences in homozygous and heterozygous genotype distributions among groups were calculated by using *χ* ^2^ test and appropriate tables. Multiple logistic regression models were applied using dominant (major allele homozygotes versus heterozygotes plus minor allele homozygotes) and recessive (major allele homozygotes plus heterozygotes versus minor allele homozygotes) models. Odds ratios (OR), 95% confidence intervals (95% C.I.), and *P* values were determined using SPSS (SPSS Inc., Chicago, IL, USA). A *P* < 0.05 was considered statistically significant.

## 3. Results

### 3.1. Analysis of the Frequencies of Ten SNPs in Our Population

Literature data have evidenced the association of the IL-10 and TGF-*β* SNPs with sporadic TAA and other cardiovascular diseases [4, 8, 12–14, 17–20]. The analysis of genotype frequencies of all SNPs examined with respect to the expected results was executed confirming that all populations were in Hardy-Weinberg equilibrium, with the exception of rs334349 genotype distribution (Table 3) in TAA patient group. The analysis of IL-10 and IL-10RB gene SNPs does not allow finding significant differences in genotype frequencies among the three populations examined (Table 3).

Comparing genotype distributions and allele frequencies of the five TGF-*β* pathway SNPs selected in our study between cases and the two control groups, significant differences were observed only for TGF-*β*2 rs900 polymorphism (see Table 4). Its frequency was significantly different in TAA patients than to both control patients (*P* = 0.047) and healthy controls (*P* = 0.0059) (Table 4). In particular, the AA genotype of TGF-*β*2 rs900 SNP had a reduced frequency in the TAA patients, which contrarily showed an increased frequency of TT genotype.

These results were confirmed by logistic regression analyses of dominant and recessive models performed between TAA patient and control groups (Table 5). Interestingly, the data obtained through comparisons between both control patients and healthy controls for dominant model and comparisons with the last group for recessive model evidenced that the presence of A allele in homo- or heterozygosis seems to be significantly protective against TAA.

Since the incidence of TAA is higher in men than in women with precisely a ratio of 3 : 1 [12], we assessed the rs900 TGF-*β* SNP frequencies according to gender. Comparing the data, we observed significant differences in genotype distribution of the rs900 SNP in women, whereas no significant differences were detected in men (Table 6). In particular, the AA genotype was significantly decreased in women affected by TAA with respect to both women of control patient group (*P* = 0.0076) and health control group (*P* = 0.0003). The TT genotype was reciprocally significantly increased (*P* = 0.0027). Thus, altogether these data emphasize cotemporally the gender related protective role of AA genotype for TAA and the increased susceptibility for TAA in individual's carriers of TT genotype (Table 6). When we perform a logistic regression analysis adjusted for gender, the significant differences of AA genotype frequency between patients and subjects of the two control groups (*P* = 0.003) and particularly between patients and healthy controls (*P* < 0.0001) were confirmed.

## 4. Discussion

Risk factors involved in developing aneurysms are similar to those for heart disease, including atherosclerosis, hypertension, smoking, advanced age, and family history. However, the lack of aneurysm-specific symptoms often renders them unnoticed until the aorta ruptures associated with significant morbidity and mortality [1, 2, 13].

TAA development proceeds as a multifactorial process influenced by both cellular and extracellular mechanisms, resulting in alterations of structure and ECM composition [1, 2]. Recent evidence underlines the deregulation of TGF-*β* signalling in ascending TAAs from syndromic (Marfan syndrome, Loeys-Dietz syndrome, and Ehlers-Danlos syndrome) and not syndromic TAA patients as well as in TAAs from cases affected by familial TAAs and dissections [14].

TGF-*β* isoforms are produced by multiple cellular types and participate in a wide array of cellular responses including proliferation, angiogenesis, differentiation, apoptosis, inflammation, and wound healing [21]. Of the three TGF-*β* isoforms, their role in matrix deposition (e.g., collagen synthesis) related to fibrotic disease is particularly well known [22]. However, some recent data have also demonstrated their involvement in unconventional pathways able to determine matrix degradation [4].

Furthermore, the TGF-*β* isoforms exhibit both overlapping and divergent properties, as evidenced principally in embryogenetic studies. In particular, TGF-*β*2 knockout mice are characterized to die perinatally and display a wide range of developmental defects, including cardiovascular, pulmonary, skeletal, ocular, inner ear, and urogenital manifestations [23–25]. Haplo-insufficient TGF-*β*2 mice have aortic root aneurysm and biochemical evidence of increased canonical and no canonical TGF-*β* signalling [26]. These observations led Maleszewska and colleagues to suggest the crucial role of TGF-*β*2 in the vascular remodelling [27]. They particularly urderlined that, in presence of a low grade chronic inflammation of the cardiac and vascular tissues, the TGF-*β*2, interacting with other cytokines as interleukin-1B, might modify and remodel vascular aorta tissues inducing endothelial cells forward mesenchymal transition (EndMT). EndMT represents a central mechanism in cardiac valve embryogenesis, which in pathological condition might determine cardiac fibrosis. Actually, immunohistochemistry analysis of TAA aneurysms demonstrated that both the media and adventitia from patients with Marfan syndrome and familial TAAs, as well as from sporadic cases with or without dissections or BAV diseases, are characterised by infiltration of inflammatory cells [27]. This inflammatory condition might contribute to the pathogenesis of TAA [3, 28–35].

In the light of these observations, we assessed the role of five genetic variants of TGF-*β* pathways (TGF-*β*1 and 2 isoforms and R1 and R2 receptors) in sporadic TAA. Interestingly, the most relevant finding of the present study allows proposing that rs900 TGF-*β*2 SNP is associated with sporadic TAA in women. On the other hand, recent reports assigned a direct or an indirect central role to TGF-*β*2 and its genetic variants in the pathogenesis of both syndromic and familial TAAs [3, 21, 28–35]. In addition, it has been reported that mutations in the TGFBRII genes deregulate the TGF-*β*2 signalling pathway involved in TAA pathogenesis [3, 28–35]. TGF-*β*2 gene mutations have been found in familial TAAs and dissections associated with mild systemic features of Marfan syndrome, Loeys-Dietz syndrome and in TAA and dissection associated with mitral valve disease [3, 28–35]. In these diseases, the TGF-*β*2 dependent EndMT might play a role. In spite of these findings, the exact role of TGF-*β*2 in TAA pathogenesis is not clear. In particular, both the loss of function genetically determined and the “paradoxical” augment in the downstream TGF-*β* signaling pathway might be important for TTA development [14].

However, to our knowledge, no literature data exist about the role of genetic variants of TGF-*β*2 pathway in sporadic TAAs. Thus, this is the first report that identified a common and functional TGF-*β*2 SNP, the rs900 SNP, as genetic risk marker for sporadic TAA. The rs900 SNP is located at the 2814 position downstream of the TGF-*β*2 gene coding region. Scanning allelic rs900 sequences for UTR structural motif using an online tool (http://itbtools.ba.itb.cnr.it/utrscan) has shown that the T allele introduces a new open reading frame ATG in the 3′UTR region of TGF-*β*2 gene that potentially might interfere with ribosomal translation. This may allow hypothesising that the rs900 T allele may interfere with the rate of protein production.

## 5. Limitations and Conclusions 

As reported above, TAAs occur most frequently in Caucasians than in other ethnic groups and they afflict men two to four times more frequently than women [4]. As consequence, our results, suggesting that rs900 TGF-*β*2 SNP might be one of genetic factors involved in the woman susceptibility for TAA, might open new perspectives for the analysis of sporadic TAA susceptibility factors and prevention. Actually these findings obtained in this relatively small study, which need certainly to be confirmed in larger populations of different genetic background, might prompt studies on gender oriented pharmacological strategies to prevent TAA development in predisposed subjects.

## Figures and Tables

**Table 1:** Demographic and clinical characteristics of TAA patients and control subjects.

Variables	AAT patients	Control patients	*P*	Control patients	*P*
Demographic characteristics	*N* = 144	*N* = 90		*N* = 168	
Age, mean (SD)	63.0 (10.7)	61.1 (5.8)	0.834	45.2 (7.4)	<0.0001
Males, number (%)	107 (74.3)	56 (62.2)	0.060	112 (66.7)	0.172
Body mass index, mean (SD)	27.0 (4.3)	26.9 (2.9)	0.898	25.8 (8.7)	0.133
TAA size and location					
Size (mm), mean (SD)	53.3 (8)	0 (0)		0 (0)	
Location, number (%)		0 (0)		0 (0)	
Ascending aorta	72 (50.0)				
Aortic bulb	16 (11.1)				
Ascending aorta and aortic bulb	56 (38.9)				
Medical history number (%)					
Aortic aneurysm familiarity	8 (5.6)	0 (0)		0 (0)	
Cardiovascular ischemic familiarity	53 (36.8)	24 (26.7)	0.089	0 (0)	
Smoking	65 (45.1)	46 (51.1)	0.420	67 (39.9)	0.360
Hypertension	114 (79.1)	28 (31.1)	<0.001	0 (0)	
Dislipidemy	33 (22.9)	14 (15.6)	0.158	0 (0)	
Diabetes mellitus	22 (15.3)	12 (13.3)	0.677	0 (0)	
Renal failure	4 (2.8)	0 (0)	0.168	0 (0)	
Dissection	16 (11.1)	0 (0)		0 (0)	
Aortic valve pathology, number (%)					
Normal	81 (56.2)	90 (100)		168 (100)	
Prolapse	19 (13.2)	0 (0)			
Vascular calcium fibrosis	45 (31.3)	0 (0)			
Aortic valve dysfunction, number (%)					
Normal	29 (20.1)	90 (100)		168 (100)	
Faint incontinence	26 (18.0)	0 (0)			
Moderate incontinence	30 (20.8)	0 (0)			
Severe incontinence	40 (27.1)	0 (0)			
Faint stenosis	1 (0.7)	0 (0)			
Moderate stenosis	2 (1.4)	0 (0)			
Severe stenosis	16 (11.1)	0 (0)			
Atherosclerosis coronary syndrome number (%)	49 (34.0)	0 (0)		0 (0)	
Drugs, number(%)					
None				168 (100)	
Beta-blockers	56 (38.9)	0 (0)		0 (0)	
Central-adrenergic agonists	23 (16.0)	0 (0)		0 (0)	
Sartans	29 (20.1)	0 (0)		0 (0)	
Calcium-channel blockers	42 (29.2)	0 (0)		0 (0)	
ACE inhibitors	59 (41.0)	14 (15.6)		0 (0)	
Antidiabetic drugs	17 (11.8)	12 (13.3)		0 (0)	
Antiaggregant drugs	46 (31.9)	28 (31.1)		0 (0)	
Antidyslipidemic drugs	32 (22.2)	0 (0)		0 (0)	
Diuretics	32 (22.2)	28 (31.1)		0 (0)	

**Table 2:** Genes, SNPs (accession number), substitutions, localization, and position investigated in the study.

Genes	SNPs	Localization	Position	Alleles
IL-10	rs1800896	Promoter	−1082	G>A
	rs1800871	Promoter	−819	C>T
	rs1800872	Promoter	−592	C>A
	rs3024496	3′UTR	Not defined	C>T

IL-10RB	rs2834167	Codifying sequencing	Codon 47	A>G

TGF-*β*1	rs1800471	Codifying sequencing	Codon 25	G>C
	rs900	3′UTR	+94862	A>T
	rs334348	3′UTR	Not defined	A>G
	rs334349	3′UTR	Not defined	A>G
	rs4522809	Intron	+3919	C>T

**Table 3:** Single nucleotide polymorphism frequencies of IL-10 pathway genes in patients affected by sporadic TAA and the two groups of controls subjects*.

SNPs	Genotypes	TAA patients *n* (%)	Control patients *n* (%)	Healthy controls *n* (%)
rs1800896	GG	17 (11.8)	7 (7.8)	21 (12.5)
	GA	66 (45.8)	44 (48.9)	67 (39.9)
	AA	61 (42.4)	39 (43.3)	80 (47.6)

rs1800871	CC	64 (44.4)	45 (50.0)	84 (50.0)
	CT	65 (45.1)	38 (42.2)	67 (39.9)
	TT	15 (10.5)	7 (7.8)	17 (10.1)

rs1800872	CC	64 (44.4)	45 (50.0)	84 (50.0)
	CA	65 (45.1)	38 (42.2)	67 (39.9)
	AA	15 (10.5)	7 (7.8)	17 (10.1)

rs3024496	CC	16 (11.1)	5 (5.5)	19 (11.3)
	CT	66 (45.8)	52 (57.8)	69 (41.1)
	TT	62 (43.1)	33 (36.7)	80 (47.6)

rs2834167	AA	67 (46.6)	35 (38.9)	91 (54.2)
	AG	66 (45.8)	44 (48.9)	60 (35.7)
	GG	11 (7.6)	11 (12.2)	17 (10.1)

*No significant differences were found comparing TAA patient genotype frequencies with control patient and healthy control groups.

**Table 4:** Genotype frequencies of TGF-*β*1 and 2 isoform and R1 and R2 receptor gene single nucleotide polymorphisms in patients affected by sporadic TAA and the two groups of controls subjects.

SNPs	Genotypes	TAA patients *n* (%)	Control patients *n* (%)	*P* value	Healthy controls *n* (%)	*P* value
rs1800471 TGF-*β*1 cod25	GG	121 (84.03)	77 (85.56)	0.499	138 (82.14)	0.849
	CG	22 (15.28)	11 (12.22)		28 (16.67)	
	CC	1 (0.69)	2 (2.22)		2 (1.19)	

rs900 TGF-*β*2 3′UTR	AA	44 (30.56)	40 (44.44)	**0.047**	77 (45.84)	0.0059*
	AT	70 (48.61)	36 (40.00)		73 (43.45)	
	TT	30 (20.83)	14 (15.56)		18 (10.71)	

rs334348 TGF-*β*R1 3′UTR	AA	92 (63.89)	56 (62.22)	0.898	106 (63.09)	0.0994
	AG	43 (29.86)	27 (30.00)		57 (33.93)	
	GG	9 (6.25)	7 (7.78)		5 (2.98)	

rs334349 TGF-*β*R1 3′UTR	GG	91 (63.20)	55 (61.11)	0.376	112 (66.67)	0.0544
	GA	39 (27.08)	30 (33.33)		51 (30.35)	
	AA	14 (9.72)	5 (5.56)		5 (2.98)	

rs4522809 TGF-*β*R2 Intron +3919	CC	36 (25.00)	24 (26.67)	0.959	44 (26.19)	0.1438
	CT	65 (45.14)	40 (44.44)		83 (49.41)	
	TT	43 (29.86)	26 (28.89)		41 (24.40)	

*The genotype distribution of rs900 TGF-*β*2 3′UTR SNP was significantly different in TAA patients when compared to both control patients and healthy controls. Allele frequencies: TAA patients: 0.549; control patients: 0.644; healthy subjects: 0.676.

**Table 5:** Multiple logistic regression analyses of dominant (major allele homozygotes versus heterozygotes plus minor allele homozygotes) and recessive (major allele homozygotes plus heterozygotes versus minor allele homozygotes) models applied to TAA patient group compared with control groups.

SNP	Model	TAA versus control patients		TAA versus healthy controls	
		OR (95% C.I.)	*P*	OR (95% C.I.)	*P*
rs1800471	Dominant	0.888 (0.452–1.858)	0.859	1.144 (0.630–2.075)	0.769
	Recessive	3.250 (0.290–36.392)	0.561	1.723 (0.154–19.210)	1.000

rs900	Dominant	0.550 (0.318–0.950)	**0.036**	0.520 (0.326–0.829)	**0.0073**
	Recessive	0.700 (0.348–1.407)	0.690	0.456 (0.242–0.859)	**0.0177**

rs334348	Dominant	1.074 (0.623–1.853)	0.889	1.035 (0.652–1.643)	0.906
	Recessive	1.265 (0.454–3.526)	0.791	0.460 (0.151–1.406)	0.181

rs334349	Dominant	1.093 (0.635–1.880)	0.782	0.858 (0.538–1.369)	0.553
	Recessive	0.546 (0.190–1.572)	0.329	0.511 (0.081–1.039)	0.0516

rs4522809	Dominant	0.917 (0.503–1.671)	0.878	0.939 (0.564–1.565)	0.897
	Recessive	0.954 (0.535–1.703)	1.000	0.758 (0.459–1.252)	0.307

**Table 6:** Statistical analysis of TGF-*β*2 rs900 genotype distributions in patients affected by TAA and in the two groups of control subjects stratified according to the gender*.

	Men			Women		
	AA	AT	TT	AA	AT	TT
TAA patients *n* (%)	36 (33.65)	54 (50.46)	17 (15.89)	8 (21.62)	16 (43.24)	13 (35.13)
Control patients *n* (%)	22 (39.29)	25 (44.64)	9 (16.07)	18 (52.94)	11 (32.35)	5 (14.71)
Healthy controls *n* (%)	43 (38.39)	56 (50.00)	13 (11.61)	34 (60.71)	17 (30.36)	5 (8.93)

	Odd ratio significance					
	Men			Women		
	AA	AT	TT	AA	AT	TT

TAA versus control patients						
OR	0.784	1.263	0.986	0.245	1.263	3.142
95% C.I.	0.401–1.531	0.660–2.418	0.408–2.382	0.087–0.689	0.660–2.418	0.990–10.071
*P* value	0.495	0.512	1.000	**0.0076**	0.512	0.059

TAA versus healthy controls						
OR	0.814	1.019	1.438	0.178	1.019	5.525
95% C.I.	0.468–1.415	0.401–1.531	0.662–3.128	0.069–0.461	0.600–1.731	1.767–17.272
*P* value	0.484	1.000	0.433	**0.0003**	1.000	**0.0027**

*Logistic regression analysis adjusted for gender confirms that the AA genotype frequency was significantly decreased and TT genotype increased in TAA women patients compared to women of the two control groups (*P* = 0.003), in particular compared to healthy controls (*P* < 0.0001).
